# Hydrostatic bearing groove multi-objective optimization of the gear ring housing interface in a straight-line conjugate internal meshing gear pump

**DOI:** 10.1038/s41598-024-62727-3

**Published:** 2024-05-28

**Authors:** Tiangui Zhang, Guishan Yan, Xianhang Liu, Beichen Ding, Guodong Feng, Chao Ai

**Affiliations:** 1 School of Intelligent Systems Engineering, Sun Yat-Sen University, Guangzhou, 510275 China; 2 The State Key Laboratory of Fluid Power and Mechatronic Systems, Hangzhou, 310000 China; 3 School of Mechanical Engineering, Yanshan University, Qinhuangdao, 066004 China; 4 School of Advanced Manufacturing, Sun Yat-sen University, Guangzhou, 510275 China

**Keywords:** Straight-line conjugate internal meshing gear pump, Hydrostatic bearing notch, Mechanical characteristics analysis, Oil film characteristic analysis, Multi-objective optimization, Mechanical engineering, Computational science

## Abstract

The lubrication performance of a straight-line conjugate internal meshing gear pump is poor under the low-speed, high-pressure operating conditions of the volumetric servo speed control system, and it is difficult to establish a full fluid lubricating oil film between the gear ring and the housing. This leads to significant wear and severe heating between the gear ring and the housing. The lubrication performance of the interface moving pair of the electro-hydraulic actuator pump gear ring housing can be improved by designing a reasonable lubrication bearing structure for the gear ring housing. In this study, a multi-field coupling multi-objective optimization model was established to improve lubrication performance and volumetric efficiency. The whole model consists of the dynamic model of the gear ring components, the fluid lubrication model of the gear ring housing interface, the oil film formation and sealing model considering the influence of temperature, and the multi-objective optimization model. The comprehensive performance of the straight-line conjugate internal meshing gear pump was verified experimentally using a test bench. The results show that the lubrication performance is improved, the mechanical loss is reduced by 31.52%, and the volumetric efficiency is increased by 4.91%.

## Introduction

**Figure 1:**
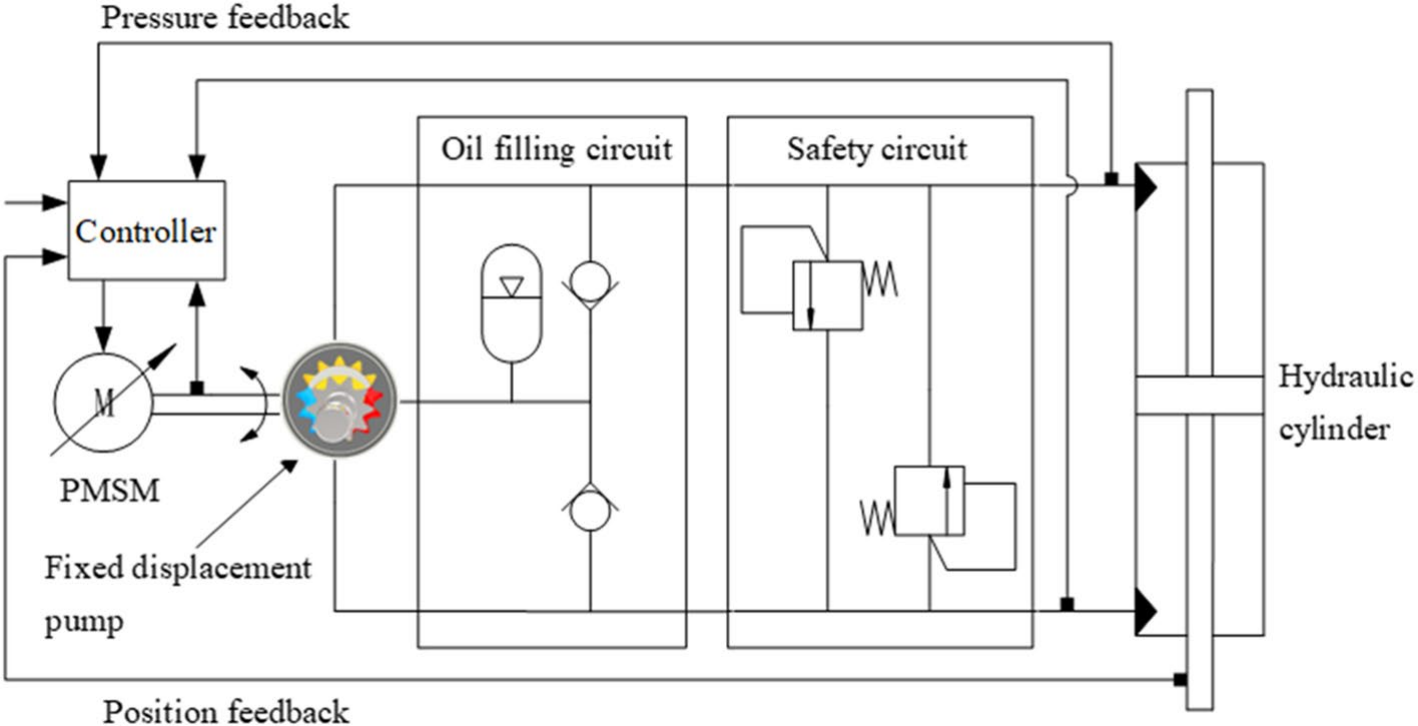
Volume servo speed control system working principle diagram.

Currently, the direct-drive electro-hydraulic actuator has good application prospects due to its high integration and energy efficiency^1^. The straight-line conjugate internal meshing gear pump is a commonly used key component in the system. Compared to the piston pump, it has the advantages of strong anti-pollution performance, simple structure, low manufacturing cost, quiet transmission, and so on^2^. The direct-drive electro-hydraulic actuator is a typical volumetric servo speed control system, and the working principle of the system is shown in Fig. 1. The volumetric servo speed control system is directly driven by the servo motor to power the fixed displacement pump. By controlling the speed of the servo motor, the output flow of the fixed displacement pump is adjusted to control the movement speed and force of the hydraulic cylinder. The system pressure, displacement, and other commands are fed back to the controller. After calculation, the controller controls the servo driver to output corresponding rotation speed commands, thus achieving closed-loop control of the system.

**Figure 2:**
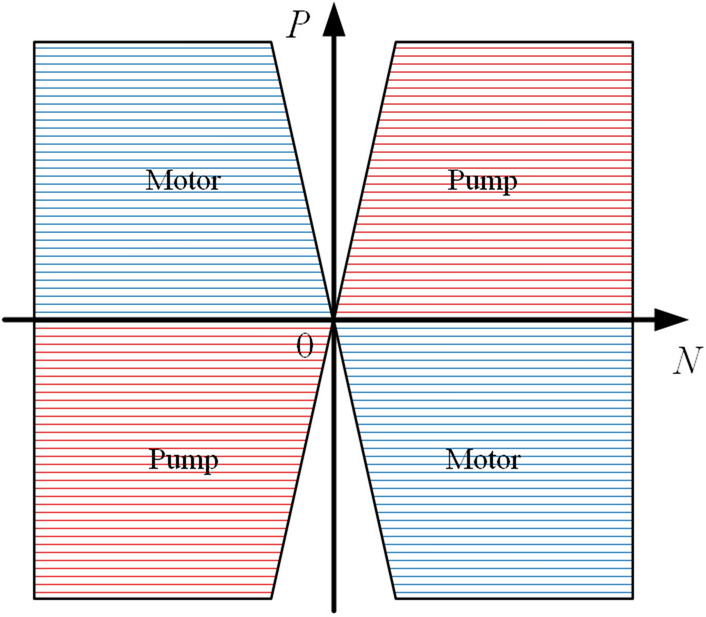
Four quadrant operating conditions of a straight-line conjugate internal meshing gear pump.

A straight-line conjugate internal meshing gear pump has the advantages of low flow pulsation, high reliability, low running noise, and compact structure, and it has a good application prospect in direct-drive electro-hydraulic actuators. A direct-drive electro-hydraulic actuator is often used for high-precision control at low speeds and under heavy loads. The speed and load conditions are variable, especially under low-speed and high-pressure conditions. It is therefore difficult to establish an ideal all-fluid lubricating oil film, which easily causes direct contact with the gear ring housing, thereby producing the gluing phenomenon, as shown in Fig. 2. In particular, the friction pair oil film at the gear ring and the housing play the roles of lubrication^3–7^, sealing^8,9^, and bearing^10,11^. Different mechanical distributions and leakage flows determine the lubrication performance and volumetric efficiency of the pump; however, the dynamic behavior of the gear ring is very complicated. For example, the gear ring meshing drive of the conjugate gear ring is affected by the time-varying meshing force and the load pressure. Under different pressures and speeds, the thickness of the oil film changes with different mechanical distributions, resulting in different tribological characteristics of the gear ring under coupling of the static pressure bearing force and dynamic pressure lubrication force. Moreover, the change in tribological properties causes a wide range of changes in the heat value of the contact interface, which further affects the film formation and leakage characteristics of the oil film^12,13^. It should be noted that the straight-line conjugate internal meshing gear pump is a strictly fixed clearance pump, and the wear caused by the gear ring and the housing under different lubrication and mechanical distribution conditions will seriously affect its reliability and efficiency^14^.

The hydrostatic bearing groove located on the surface of the shell is considered a feasible choice for overcoming the radial unbalanced force of the pump^15^. Moreover, it can improve the dynamic and static lubrication performance of the gear ring and reduce the friction and wear of the pump caused by an uneven force distribution. Enhancement of the hydrodynamic lubrication and support performance depends on factors such as the geometry of the notch, opening position, and opening angle^16–18^. Therefore, hydrostatic bearing notch technology is applied to improve the mechanical distribution and lubrication performance of the pump; however, the geometric parameters of the hydrostatic bearing slot are sensitive to changes in dynamic characteristics and working conditions, and improperly designed parameters will have adverse effects on lubrication and support performance^19^. Most studies in the published research on hydrostatic bearing technology primarily focus on bearings^20,21^ and mechanical seals^22,23^.

At low speed, the oil film distribution of the straight-line conjugate internal meshing gear pump changes with time, and the force of the gear ring changes significantly under different working conditions, which is very different from the force and lubrication characteristics analysis of static pressure bearings. There are currently limited studies characterizing the hydrostatic bearing notch of a straight-line conjugate internal meshing gear pump, and research on static lubrication characteristics mostly focuses on applying the optimal static support effect to the surface contact friction pair. The Xie Z system studies the influence of varying surface roughness and bearing radial clearance on lubrication performance and lubrication state transformation. An improved lubrication model for eccentric dislocations is proposed^24^. In view of the influence of geometric parameters and the shape of the lubricated surface on friction and wear, Xu and Yufu proposed an elliptical concave anti-friction geometrical structure to reduce friction under lubrication^25^. Zhang F studied the effect of roughness and surface micro-pit texture on the oil film sealing performance, established a numerical model of oil seal mixed lubrication in the sealing area, and obtained the mapping relationship between the texture shape, roughness, rotational speed, and other parameters on oil film thickness and friction torque^26^. These studies provide the basis for optimizing the hydrostatic bearing notch at the ring/shell interface; however, the two issues that follow remain to be resolved.

First, the force of the gear ring during movement is complex and changeable. The gear ring transmission meshing force, high pressure on the gear ring, hydrostatic bearing force, and dynamic pressure bearing capacity change with the load pressure and input speed. Song Wei proposed a method to determine the envelope angle of the pressure cavity based on a graphical model and obtained the law of the variation of the envelope angle of the pressure oil cavity with the rotation of the gear^27,28^. Du Ruilong studied the factors that formed the pressure field of the lubrication film between the tooth ring and the friction pair of the shell and obtained the law of the influence of different working conditions and radial clearance on the minimum lubrication film thickness of the friction pair^3^. To elucidate the influence of gear compression and meshing force, Zhang Jianzhuo proposed a hydrostatic bearing structure to improve the friction performance of the internal meshing gear pump under high speed and high pressure^29^. Therefore, it is necessary to establish a mechanical distribution prediction model to obtain the numerical simulation fluid pressure interface of the oil film lubrication, sealing, and bearing properties.

The lubricating film thickness, geometry, and lubrication boundary conditions at the interface between the tooth ring and shell of a straight-line conjugate internal meshing gear pump exhibit the characteristics of a time-varying and non-uniform distribution. Additionally, some effects of the hydrostatic bearing notch on the straight-line conjugate internal meshing gear pump are contradictory. Many researchers have made efforts to apply surface-grooving technology to the friction pairs of hydraulic pumps^30–32^. The bearing capacity of dynamic pressure lubrication decreases under low-speed and high-pressure conditions^33^. Only by increasing the static pressure bearing force to improve the bearing capacity of the oil film will the thickness of the oil film increase, and the leakage flow will increase rapidly^34,35^. While the mechanical efficiency increases, the volumetric efficiency decreases. Therefore, the objective of optimization is to improve the bearing capacity under the combined action of static pressure bearing and dynamic pressure lubrication and reduce the leakage flow at the interface of the gear ring housing. Further, it is necessary to establish a multi-objective optimization model to address the contradictory effects of the hydrostatic bearing notch and improve the overall performance of the pump.

In this study, a multi-objective optimization model for the static support notch of a straight-line conjugate internal meshing gear pump housing is presented. The dynamic distribution prediction model of the gear ring and the hydrodynamic lubrication model of the friction interface of the gear ring housing were established. In the multi-objective optimization model, the bearing capacity of the oil film, mechanical efficiency, and leakage flow between the gear ring and the housing were all regarded as optimization objectives. The optimal Pareto solution set in the shape of the hydrostatic bearing notch was obtained by the non-dominated genetic differential optimization method. The distribution laws of the four forces under different working conditions were analyzed by numerical simulation. Finally, the improved pump prototype was evaluated on the test bench of a straight-line conjugate internal meshing gear pump. The experimental results show that the optimized hydrostatic bearing notch can improve the lubrication state of the interface friction pair of the gear ring housing under low speed and high pressure conditions, effectively improve the mechanical efficiency of the pump, and improve the overall efficiency.

## Multi-objective optimization model of a hydrostatic bearing notch

### Mechanical model of a gear ring

To avoid the long-term mixed friction caused by the serious eccentricity of the gear ring or the contact dry friction caused by the difficult formation of a lubricating oil film, which eventually leads to wear failure of the component, dynamic analysis of the gear ring assembly was carried out.

**Figure 3:**
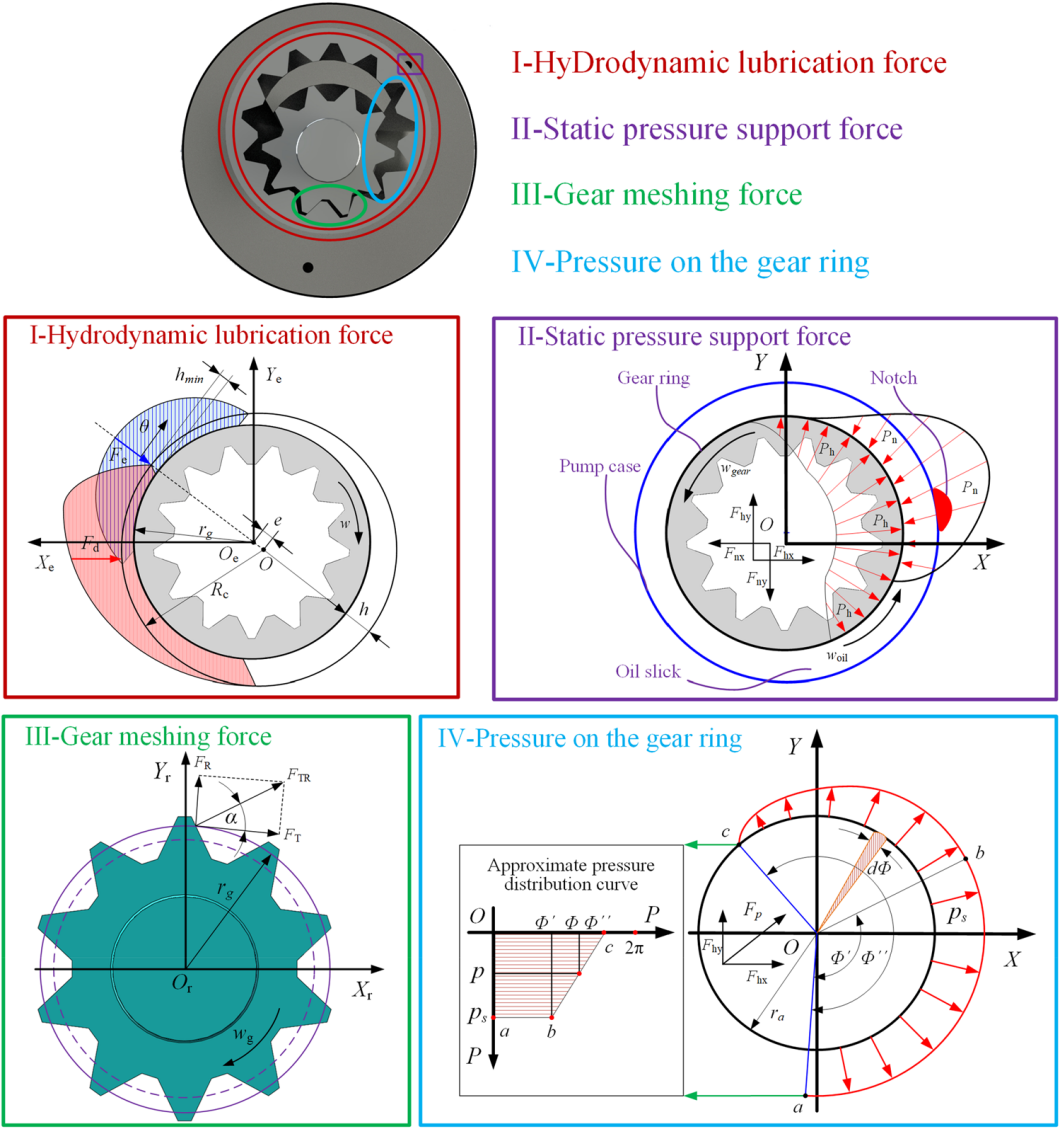
Mechanical characteristics analysis of a straight-line conjugate internal meshing gear pump.

The coordinate system *O-XY,* the additional coordinate system *O*_r_*-X*_r_*Y*_r_*,* and the additional coordinate system *O*_e_-*X*_e_*Y*_e_ are established with the center of the outer surface of the gear ring as the origin. The coordinate system definition is shown in Fig. 3. The resulting force *F*_res_ during the motion of the gear ring consists of four parts: the radial meshing force *F*_R_ of the gear ring transmission, the hydraulic pressure *F*_p_ of the high-pressure side oil on the gear ring, the hydrostatic bearing force *F*_e_, and the dynamic pressure lubrication capacity *F*_d_.

1 $$F_{res} + F_{R} + F_{d} + F_{e} + F_{p} = 0$$

Gear ring drive meshing is described as follows:

2 $$F_{R} = F_{TR} \sin \alpha = \frac{{\Delta pV_{B} }}{{2\pi \eta_{{{\rm B}m}} r_{g} }}\tan \alpha$$

where *F*_TR_ is the meshing drive force of the gear ring, *V*_B_ is the geometric displacement of the hydraulic pump, *η*_Bm_ is the mechanical efficiency of the hydraulic pump, *α* is the meshing drive pressure angle, and *r*_g_ is the radius of the gear division circle.

The force of high pressure on the gear ring and the meshing force between the gear ring are the active forces on the gear ring. The force of high pressure on the gear ring is much larger than the meshing force, so we ignore it in the simulation study for the convenience of calculation.

The hydraulic pressure generated by the high-pressure side oil on the gear ring is determined by the following:

If a small area with an angle of *d*φ and a width of *B* is taken from the top circle of the gear ring, the component force *dF*_hx_, *dF*_hy_ in the direction of the *x* and *y* axes is also exerted on this small area.

3 $$\left\{ \begin{gathered} dF_{hx} = p_{s} Br_{a} \cos \phi d\phi \hfill \\ dF_{hy} = p_{s} Br_{a} \sin \phi d\phi \hfill \\ \end{gathered} \right.$$

4 $$F_{P} = \sqrt {F_{hy}^{2} + F_{hx}^{2} }$$

5 $$\left\{ \begin{gathered} F_{hx} = Br_{a} p_{s} \frac{\cos \phi ^{\prime \prime} - \cos \phi^{ \prime} }{{\phi ^{ \prime \prime} - \phi ^ \prime }} \hfill \\ F_{{h{\text{y}}}} = Br_{a} p_{s} \left( {\frac{\sin \phi ^{\prime \prime} - \sin \phi ^\prime }{{\phi^{ \prime \prime} - \phi ^\prime }} - 1} \right) \hfill \\ \end{gathered} \right.$$

where *F*_hx_ and *F*_hy_ are the hydraulic component forces of the inner gear ring in the *x* and *y* axis directions, respectively; *r*_a_ is the radius of the tooth ring division circle; *p*_s_ is the liquid pressure in the oil pressure area; *Φ*' is the envelope angle of the section connected between the tooth ring and the low pressure chamber; *Φ*" is the sum of the envelope angle of the transition zone between the low pressure chamber and the high and low pressure chamber of the inner gear ring; and *B* is the tooth width.

**Figure 4:**
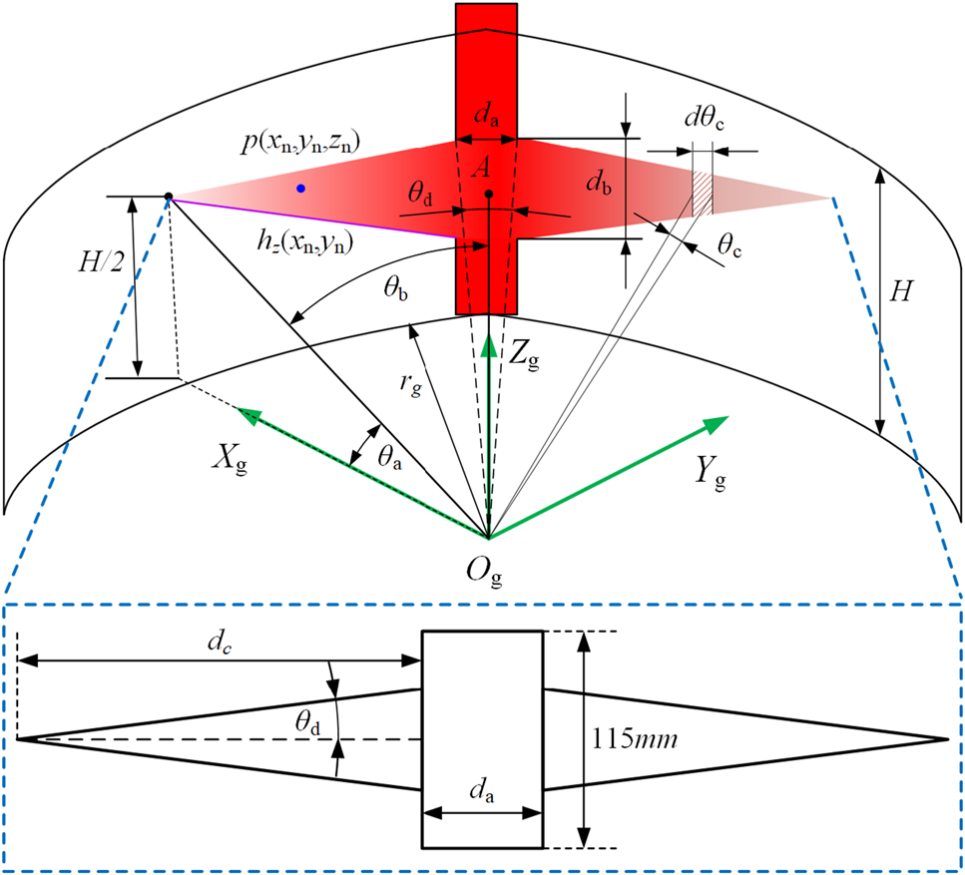
Straight-line conjugate internal meshing gear pump tooth ring housing interface notch.

The structure of the housing static pressure bearing groove of the internal meshing gear pump is shown in Fig. 4.

Due to the influence of dynamic pressure lubrication and leakage, there is a large difference between the pressure at the boundary of the hydrostatic support groove and that at the inlet. We express the pressure at different positions as *p*(*x*_n_,*y*_n_,*z*_n_), and the points with the same *z* coordinate in the hydrostatic support groove are approximated as the same pressure. The hydrostatic support force is expressed as follows:

6 $$F_{e} = p_{in} H \cdot d_{a} + \int_{0}^{{\theta_{b} - \frac{{\theta_{d} }}{2}}} {\left[ {H - 2h_{z} \left( {x_{n} ,y_{n} } \right)} \right]} \cdot p\left( {x_{n} ,y_{n} ,x_{n} } \right)d\theta_{c}$$

where *p*_in_ is the inlet pressure, where the default pressure in the rectangular groove is the inlet pressure; *H* is the thickness of the tooth ring; *d*_a_ is the width of the rectangular groove; *θ*_b_ is the angle from the center of the hydrostatic support groove to the edge of the included angle; *θ*_d_ is the angle corresponding to the rectangular groove; *h*_z_(*x*_n_,*y*_n_) is the function of the height of the blue point in the Fig. 4; and *θ*_c_ is the length of a micro-circle on the selected hydrostatic support groove.

### Lubrication model of the oil film at the interface of the gear ring housing

**Figure 5:**
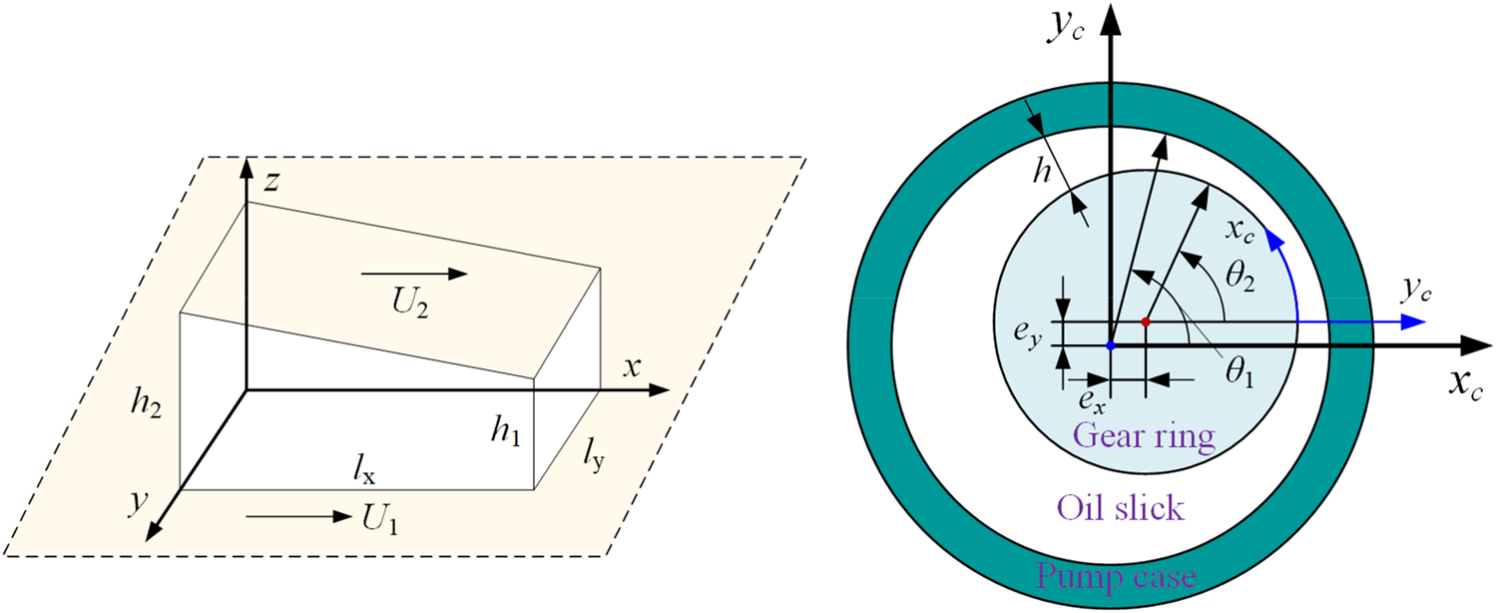
Model diagram of the gear ring housing interface lubricating oil film in a straight-line conjugate internal meshing gear pump.

The flow state of the fluid between the tooth ring and the shell can be simplified into a two-dimensional fluid lubrication problem. The schematic diagram of the lubricating oil film model is shown in Fig. 5. The basic equations for solving fluid lubrication include the continuity equation, the equilibrium equation, and the constitutive equation.

Continuity equation

7 $$\frac{\partial \rho }{{\partial t}} + \frac{{\partial \left( {\rho u} \right)}}{\partial x} + \frac{{\partial \left( {\rho v} \right)}}{\partial y} + \frac{{\partial \left( {\rho w} \right)}}{\partial z} = 0$$

Equilibrium equation

8 $$\frac{\partial p}{{\partial x}} = \frac{{\partial \tau_{xz} }}{\partial z} \, , \, \frac{\partial p}{{\partial y}} = \frac{{\partial \tau_{yz} }}{\partial z} \, , \, \frac{\partial p}{{\partial z}} = 0$$

Constitutive equation

9 $$\tau_{zx} = f\left( {\frac{du}{{dz}}} \right) \, , \, \tau_{zy} = f\left( {\frac{dv}{{dz}}} \right)$$

In the Eqs. 7, 8, 9, *x*, *y*, and *z* are the coordinate directions; *u*, *v*, and *w* are the flow velocities of the fluid along the three coordinate axes *x*, *y*, and *z*, respectively; *ρ* is the fluid density; *p* is the fluid pressure; and *τ*_zx_ and *τ*_zy_ are the shear stresses in the *x* and *y* directions on the normal vector plane with the *z* axis, respectively.

The eccentricity between the inner gear ring and the housing is expressed as follows:

10 $$\left( {e_{x} ,e_{y} } \right) = \left( {\left( {e_{2x} - e_{1x} } \right)\frac{{\hat{y}}}{{l_{gap} }} + e_{1x} ,\left( {e_{2y} - e_{1y} } \right)\frac{{\hat{y}}}{{l_{gap} }} + e_{1y} } \right)$$

where y is the axial position of the plane, and l is the length of the oil film of the friction pair.

The oil film thickness *h,* calculated at the point (*x*,*y*) of the oil film in the coordinate system *o*-*xyz*, is expressed as follows:

11 $$h\left( {\hat{x},\hat{y}} \right) = \sqrt {\left( {r_{c} \cos \theta_{1} - e_{x} } \right)^{2} + \left( {r_{c} \sin \theta_{1} - e_{z} } \right)^{2} } - r_{p}$$

where *r*_c_ is the radius of the inner surface of the shell, and *r*_p_ is the radius of the tooth ring.

Since the thickness of the oil film between the gear ring and the housing is different from the radius of the plunger by several orders of magnitude, Eq. 12 can be considered valid.

12 $$\theta_{1} \approx \theta_{2} = \frac{{\hat{x}}}{{r_{p} }}$$

13 $$h\left( {\hat{x},\hat{y}} \right) = \sqrt {\left( {r_{c} \cos \left( {\frac{{\hat{x}}}{{r_{p} }}} \right) - e_{x} } \right)^{2} + \left( {r_{c} \sin \left( {\frac{{\hat{x}}}{{r_{p} }}} \right) - e_{z} } \right)^{2} } - r_{p}$$

Equations describing lubrication are usually reduced to ordinary differential equations. The model of hydrodynamic lubrication with the same density and viscosity can be simplified as follows:

14 $$\begin{gathered} \frac{\partial }{\partial x}\left[ {h^{2} \left( {\hat{x},\hat{y}} \right)\left( {\frac{{h\left( {\hat{x},\hat{y}} \right)}}{\eta }\frac{\partial p}{{\partial x}}} \right)^{\frac{1}{n}} } \right] + \frac{\partial }{\partial y}\left[ {h^{2} \left( {\hat{x},\hat{y}} \right)\left( {\frac{{h\left( {\hat{x},\hat{y}} \right)}}{\eta }\frac{\partial p}{{\partial y}}} \right)^{\frac{1}{n}} } \right] \hfill \\ = \frac{{2^{{1 + \frac{1}{n}}} \left( {2n + 1} \right)\mathop u\limits^{\_} }}{n}\frac{{\partial h\left( {\hat{x},\hat{y}} \right)}}{\partial x} \hfill \\ \end{gathered}$$

where the velocity in the *y* direction is *v*_1_ = *v*_2_ = 0 m/s.

Equation 15 sets the pressure boundary conditions for dynamic lubrication.

15 $$\left\{ \begin{gathered} p|_{x = 0} = 0 \, , \, p|_{{x = l_{x} }} = 0 \hfill \\ p|_{y = 0} = 0 \, , \, p|_{{y = l_{y} }} = 0 \hfill \\ \end{gathered} \right.$$

The dimensionless quantity is introduced into the hydrodynamic lubrication equation.

16 $$\eta^{ * } = \frac{\eta }{{\eta_{0} }},P = \frac{p}{{p_{H} }},H = \frac{h}{{h_{0} }},X = \frac{x}{{l_{x} }},Y = X = \frac{y}{{l_{y} }},Z = \frac{z}{h},U = \frac{u}{{u_{0} }}$$

where *η*_0_ is the initial viscosity of the anti-wear hydraulic oil, *p*_H_ is the initial contact stress, *h* is the thickness of the oil film, *l* is the length of the dynamic pressure lubrication part of the gear ring, and *h*_0_ is the initial oil film thickness.

Assuming that the viscosity and density of hydraulic oil are constant, the lubrication equation after the unified dimension is expressed as follows:

17 $$\frac{\partial }{\partial X}\left( {\varepsilon_{x} \frac{\partial P}{{\partial X}}} \right) + \frac{\partial }{\partial Y}\left( {\varepsilon_{y} \frac{\partial P}{{\partial Y}}} \right) = \frac{\partial H}{{\partial X}}$$

where $$\upvarepsilon _{x} = k_{0} \rho^{*} H^{{2 + \frac{1}{n}}} (\frac{\partial P}{{\partial X}})^{{\frac{1}{n} - 1}}$$, $$\upvarepsilon _{y} = k_{0} \rho^{*} H^{{2 + \frac{1}{n}}} (\frac{\partial P}{{\partial Y}})^{{\frac{1}{n} - 1}}$$, and $$k_{0} = \frac{n}{2n + 1}\frac{{h_{0} }}{{2\overline{u} }}\left( {\frac{{h_{0} p_{H} }}{{2\eta_{0} l_{x} }}} \right)()^{\frac{1}{n}}$$.

The hydrodynamic lubrication boundary conditions after a unified dimension are as follows:

18 $$\left\{ \begin{gathered} P|_{X = 0} = 0,P|_{X = 1} = 0 \hfill \\ P|_{Y = 0} = 0,P|_{Y = 1} = 0 \hfill \\ \end{gathered} \right.$$

Because the dimensionless lubrication equation contains differential terms, it needs to be discretized for a numerical solution.

Discretization of Eq. 17 yields:

19 $$\frac{{\varepsilon_{i - 1/2,j} P_{i - 1,j} + \varepsilon_{i + 1/2,j} P_{i + 1,j} + \varepsilon_{i,j - 1/2} P_{i,j - 1} + \varepsilon_{i,j + 1/2} P_{i,j + 1} - \varepsilon_{0} P_{i,j} }}{{\vartriangle X^{2} }} = \frac{{H_{ij} - H_{i - 1,j} }}{\vartriangle X}$$

where $\upvarepsilon _{i \pm 1/2,j} = \frac{1}{2}\left[ {\left( {\upvarepsilon _{X} } \right)_{i,j} + \left( {\upvarepsilon _{X} } \right)_{i \pm 1,j} } \right]$; $\upvarepsilon _{i,j \pm 1/2} = \frac{1}{2}\left[ {\left( {\upvarepsilon _{X} } \right)_{i,j} + \left( {\upvarepsilon _{X} } \right)_{i,j \pm 1} } \right]$;$\upvarepsilon _{0} =\upvarepsilon _{i + 1/2,j} +\upvarepsilon _{i - 1/2,j} +$ $\upvarepsilon _{i,j + 1/2} +\upvarepsilon _{i,j - 1/2}$; and $\Delta X = X_{{\text{i}}} - X_{i - 1}$ $H_{ij} ,P_{ij}$ are the film thickness and pressure values of node $\left( {i,j} \right)$.

This yields the following:

20 $$P_{ij} = \frac{{r_{ij} - \vartriangle X\left( {H_{ij} - H_{i - 1,j} } \right)}}{{\varepsilon_{0} }}$$

where $$r_{ij} =\upvarepsilon _{i - 1/2,j} P_{i - 1,j} +\upvarepsilon _{i + 1/2,j} P_{i + 1,j} +\upvarepsilon _{i,j - 1/2} P_{i,j - 1} +\upvarepsilon _{i,j + 1/2} P_{i,j + 1}$$.

The film thickness equation of hydrodynamic lubrication is known, and the pressure distribution of the lubricating oil film is obtained by numerical iteration of Eq. 20. First, the initial pressure distribution is given, and the lubrication equation is used to solve the new pressure distribution until the two pressure differences meet the convergence condition.

The leakage flow of the outer wall of the gear ring and the inner wall of the shell is expressed as follows:

21 $$Q_{\delta } = \frac{B\Delta p}{{12\mu L}}\delta^{3} - \frac{Bv}{2}\delta$$

The friction shear stress between the outer wall of the gear ring and the inner wall of the shell is expressed as follows:

22 $$\tau_{\delta } = \frac{\Delta p\delta }{{4\pi r_{g} }} + \frac{{\mu \pi Nr_{g} }}{30\delta }$$

The viscous friction force between the outer wall of the gear ring and the inner wall of the shell is expressed as follows:

23 $$F_{h\delta } = \tau_{\delta } A = \frac{\Delta p\delta B}{2} + \frac{{\mu \pi^{2} BNr_{g}^{2} }}{15\delta }$$

The viscous friction loss between the outer wall of the gear ring and the inner wall of the shell is expressed as follows:

24 $$\Delta P_{h\delta } = \frac{1}{30}B\pi Nr_{g} \left( {\frac{\Delta p\delta }{2} + \frac{{\mu N\pi^{2} r_{g}^{2} }}{15\delta }} \right)$$

where *δ* is the gap between the tooth ring and the inner wall of the shell, *L* is the length of the gap along the leakage direction *δ*, *v* is the linear velocity of the outer circle of the tooth ring, *μ* is the dynamic viscosity of the oil, and *N* is the speed of the tooth ring.

### Multi-objective optimization simulation model

**Figure 6:**
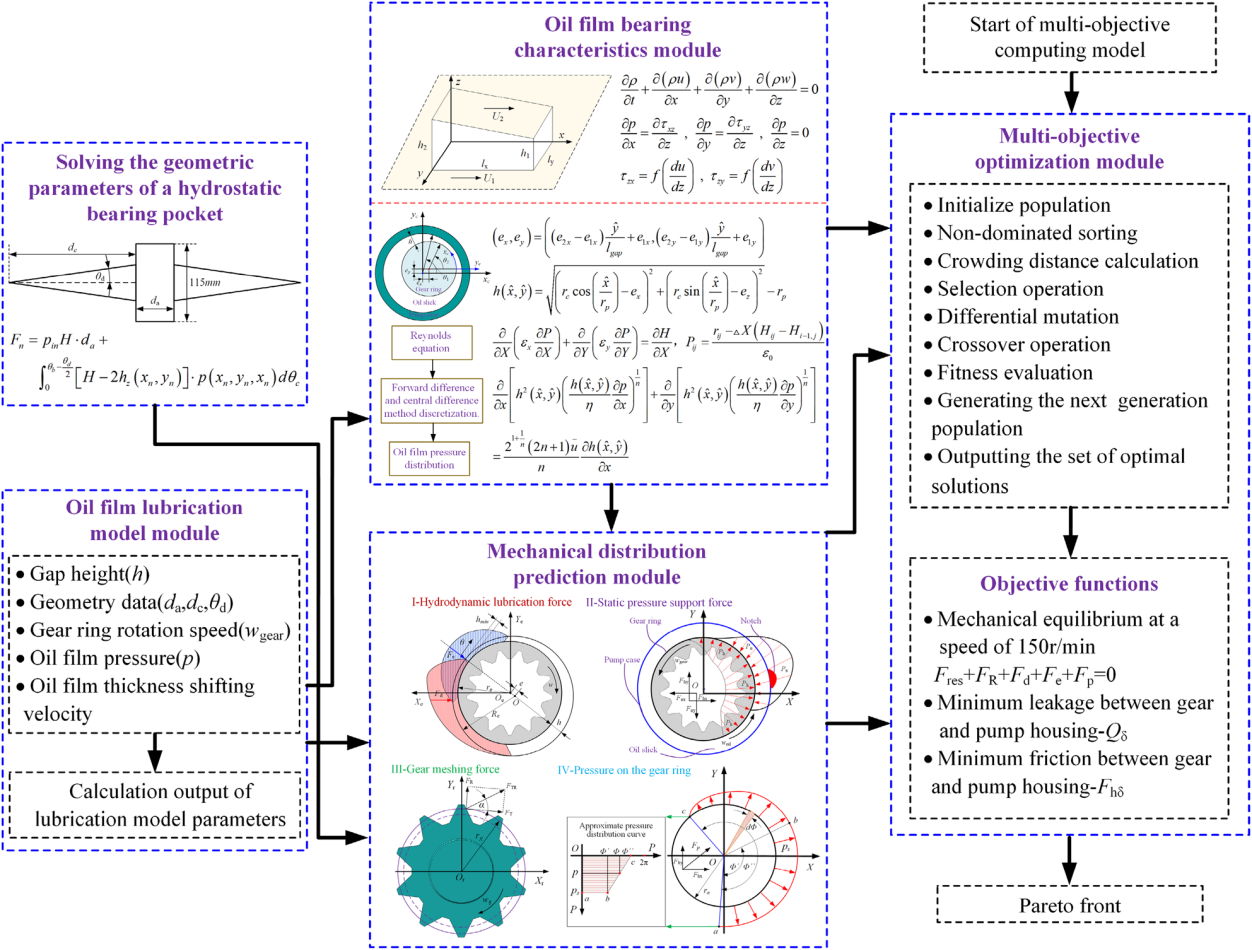
Multi-objective optimization flowchart of the tooth ring housing interface.

The multi-objective optimization model of the tooth ring housing interface mainly includes three parts: the oil film lubrication and load characteristics module, the mechanical distribution prediction module, and the multi-objective optimization algorithm. The non-dominated genetic differential evolution multi-objective optimization algorithm (NSDE) is an effective method to solve multi-objective optimization problems. The mathematical model expression is as follows:

25 $$SDE = \left( {D,NP,G,F,CR,T} \right)$$

where *D* represents the population dimension, *NP* represents the population number, *G* represents the maximum number of iterations, *F* represents the mutation operator, *CR* represents the crossover operator, and *T* represents the iteration termination condition. The model should consider not only the mechanical characteristics of the gear ring but also the influence of the parameters of the static support notch on the mechanical characteristics. The oil film support model is input to the multi-objective optimization model for producing an iterative solution. The simulation calculation process is shown in Fig. 6.

In the simulation model, the simulation parameters of the lubricating oil film model are shown in Table 1, and the simulation parameters of the NSDE are shown in Table 2.

**Table 1:** Lubricating oil film model numerical calculation conditions.

Symbol	Value	Unit
Plane 1 moving speed, *u*_1_	0	m/s
Plane 2 moving speed, *u*_2_	0.287	m/s
Power function exponent, *n*	0.9	–
Height 1, *h*_1_	0.025	mm
Height 2, *h*_2_	0.035	mm
Axis *x*,*y* direction Length projection, *l*_x_ = *l*_y_	0.005	m

**Table 2:** Multi-objective optimization model simulation parameters.

Symbol	Value
Spatial dimension, *D*	4
Population size, *NP*	100
Original scaling factor, *F*_0_	0.5
Cross probability, *CR*	0.9
Maximum iterations, *N*	500

In the simulation model, the parameters of the straight-line conjugate internal meshing gear pump, operating conditions, and hydraulic oil parameters are as shown in Table 3.

**Table 3:** Straight-line conjugate internal meshing gear pump parameters and operating condition parameters.

Argument	Value	Unit
Rotational speed, *n*_p_	150	r/min
Pump outlet pressure, *p*_in_	3–18	MPa
Pump inlet pressure, *p*_out_	0.1	MPa
Gear ring outer wall radius, *r*_g_	114.95	mm
Shell inner radius, *R*_c_	115.05	mm
Hydrostatic support groove depth	5	μm
Pump displacement, *V*	63.5	ml/r
Oil temperature, *T*	40	°C
Oil density, *ρ*_o_	850	kg/m^3^
Ring thickness	34.05	mm
Ring teeth number	13	–
Teeth number	9	–
Oil dynamic viscosity	46	mm^2^/s

## Simulation results and discussion

**Figure 7:**
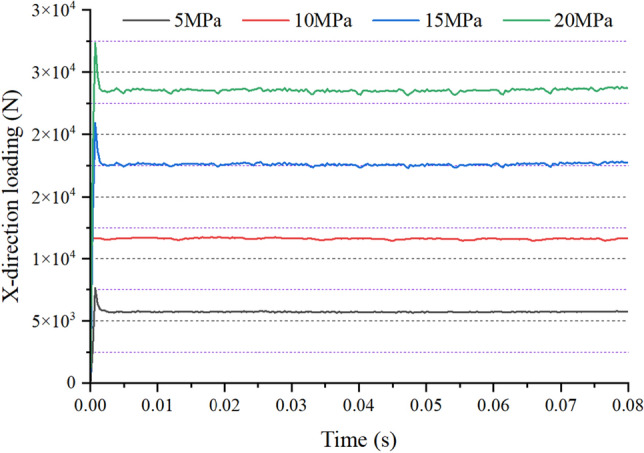
X-direction force of high-pressure oil on the gear ring.

**Figure 8:**
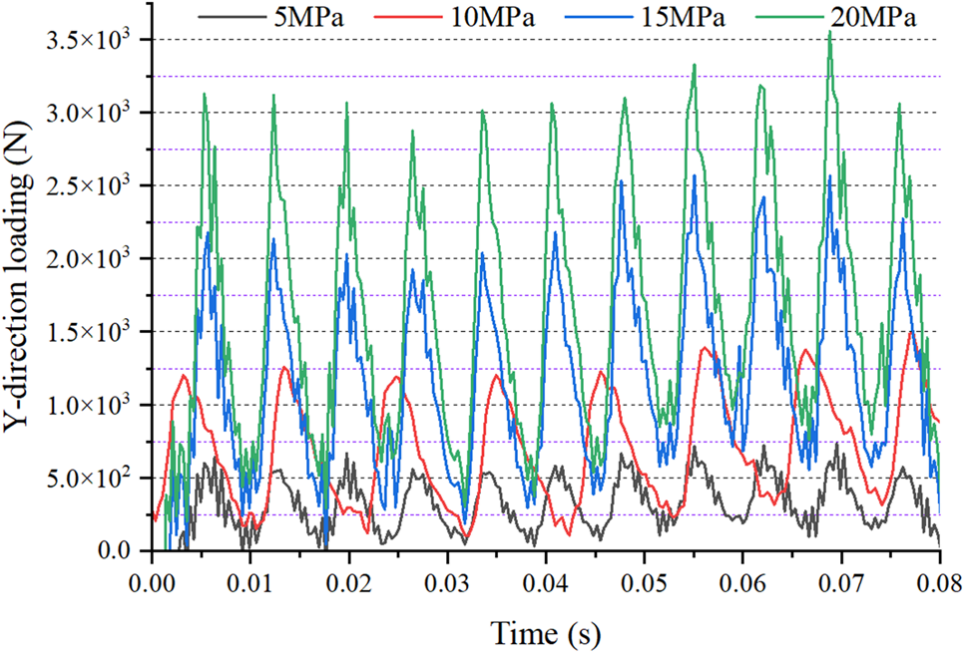
Y-direction force of high-pressure oil on the gear ring.

As shown in Figs. 7 and 8, the force of the gear ring under different pressure conditions is simulated and analyzed. The force in the X direction fluctuates slightly with the rotation of the gear ring, including 178.4 N for 5 MPa, 346.6 N for 10 MPa, 596.4 N for 15 MPa, and 803.3 N for 20 MPa. With the increase in pressure, the force presented a linear increase. The force in the Y direction changes greatly with the rotation of the gear ring. The pressure fluctuation of 5 MPa is 736.2 N, that of 10 MPa is 1760.6 N, that of 15 MPa is 2623.2 N, and that of 20 MPa is 3620.1 N. The mechanical simulation results are input to the multi-objective optimization simulation model.

**Figure 9:**
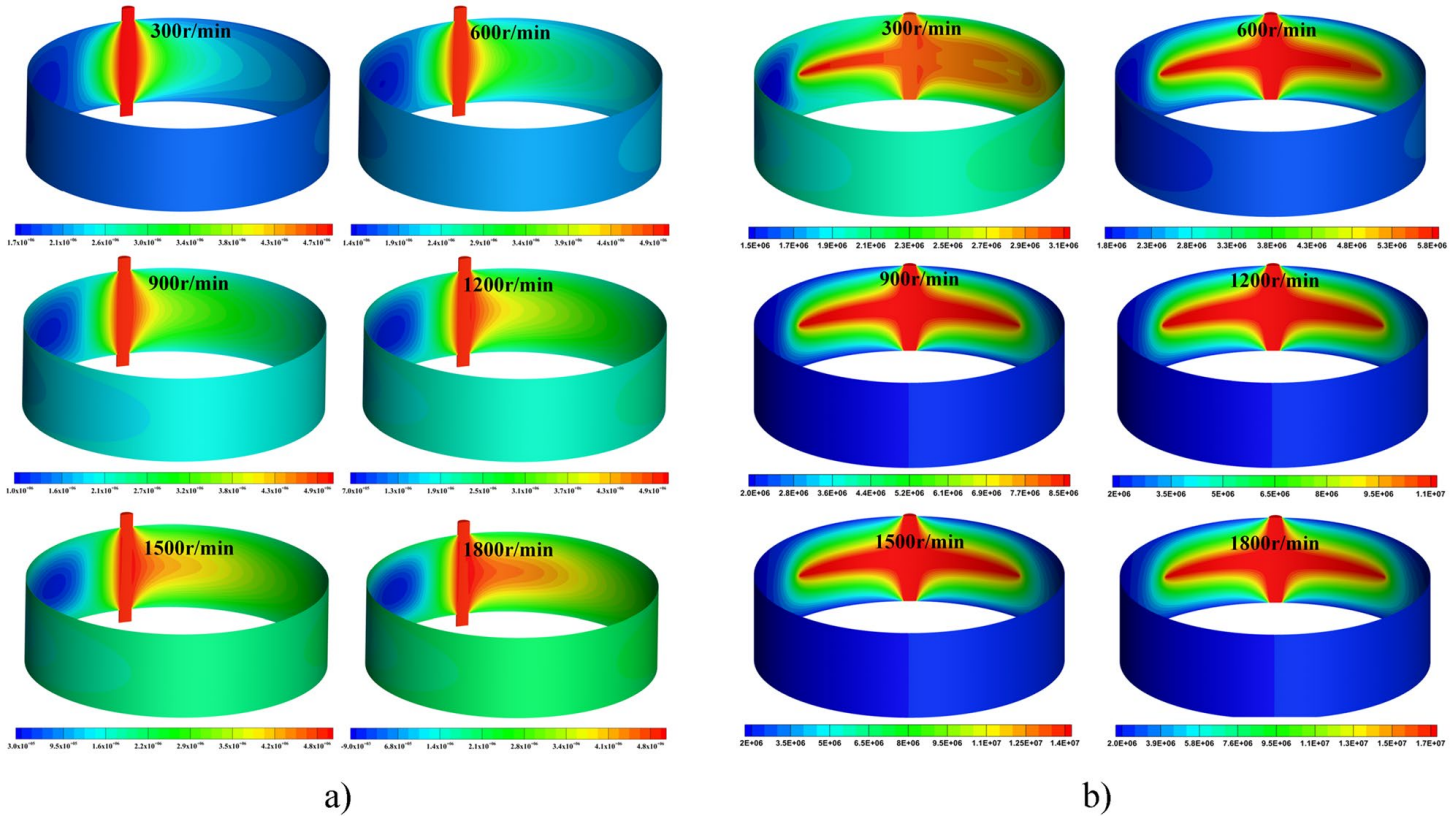
Load bearing pressure optimization of the front and rear notches. (**a**) Optimized front notch load. (**b**) Optimized slot load.

As shown in Fig. 9, the dynamic pressure bearing effect on the interface of the optimized front gear ring housing increases from 300 r/min to 1800 r/min, and the maximum pressure increases from 4.7 MPa to 4.9 MPa, which then decreases from 4.9 MPa to 4.8 MPa. The dynamic pressure lubrication effect first increases and then decreases. The negative pressure effect on the other side of the notch is also enhanced correspondingly; the pressure changes rapidly, and the bubble easily precipitates. After optimization, the speed of the bearing effect on the interface of the gear ring housing increased from 300 r/min to 1800 r/min, and the dynamic lubrication effect increased sharply by 5.48 times. The results of the dynamic lubrication simulation are input to the multi-objective optimization simulation model.

**Figure 10:**
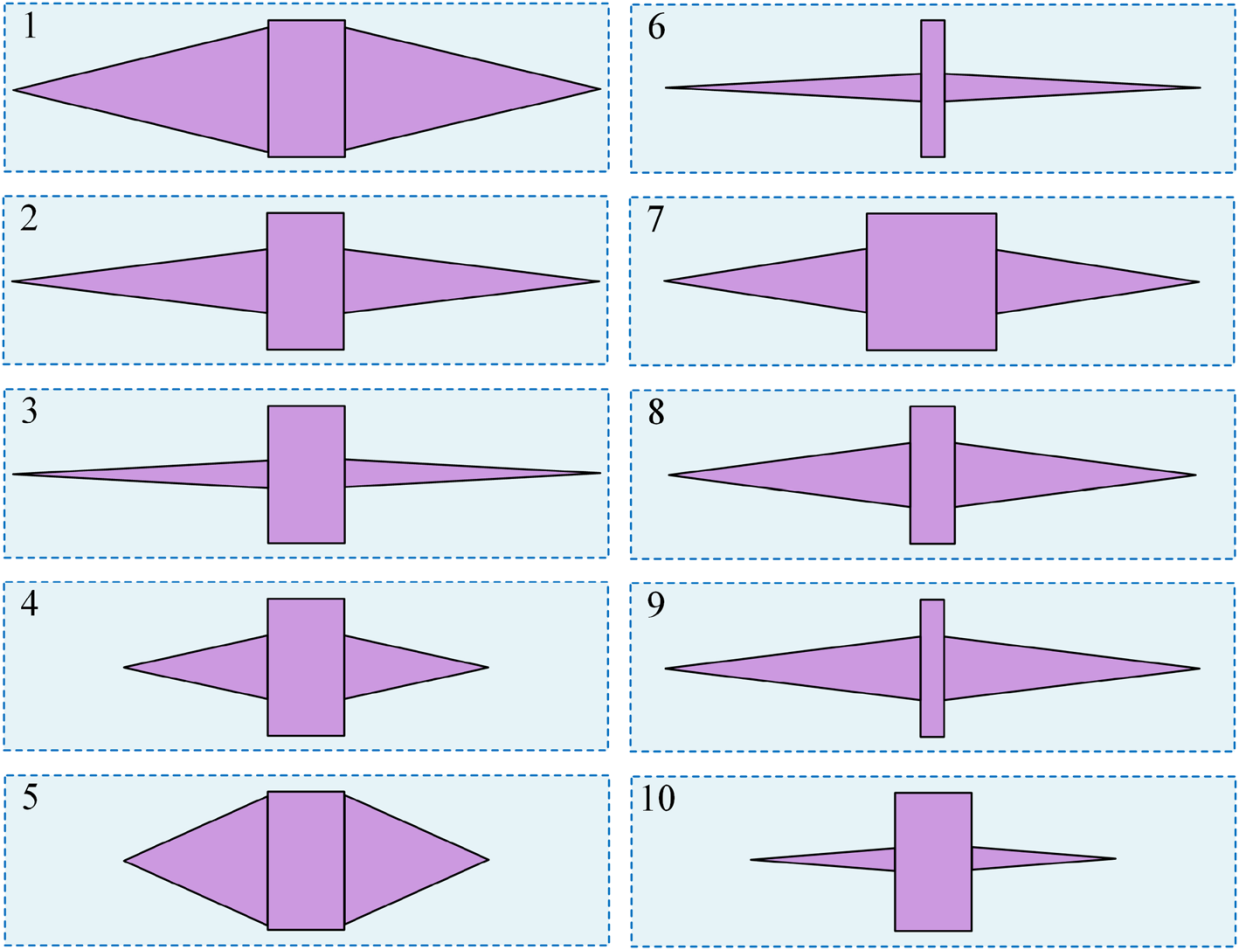
Geometry of the optimal solution of the hydrostatic bearing notch.

Ten optimal solutions are obtained through the non-dominated genetic differential evolution multi-objective optimization algorithm, and the geometry of the optimal solution is shown in Fig. 10. The geometric parameters of optimal solution 1 are (*d*_a_:64.14, *θ*_d_:27.42, *d*_c_:39.41), those of 2 are (*d*_a_:64.14, *θ*_d_:14.24, *d*_c_:39.31), those of 3 are (*d*_a_:64.14, *θ*_d_:6.2, *d*_c_:39.30), those of 4 are (*d*_a_:64.14, *θ*_d_:25.17, *d*_c_:22.04), those of 5 are (*d*_a_:64.14, *θ*_d_:47.98, *d*_c_:22.18), those of 6 are (*d*_a_:19.66, *θ*_d_:26.17, *d*_c_:39.19), those of 7 are (*d*_a_:108.19, *θ*_d_:18.08, *d*_c_:31.08), those of 8 are (*d*_a_:37.30, *θ*_d_:15.18, *d*_c_:37.01), those of 9 are (*d*_a_:19.66, *θ*_d_:14.33, *d*_c_:39.11), and those of 10 are (*d*_a_:64.14, *θ*_d_:8.95, *d*_c_:22.01).

**Figure 11:**
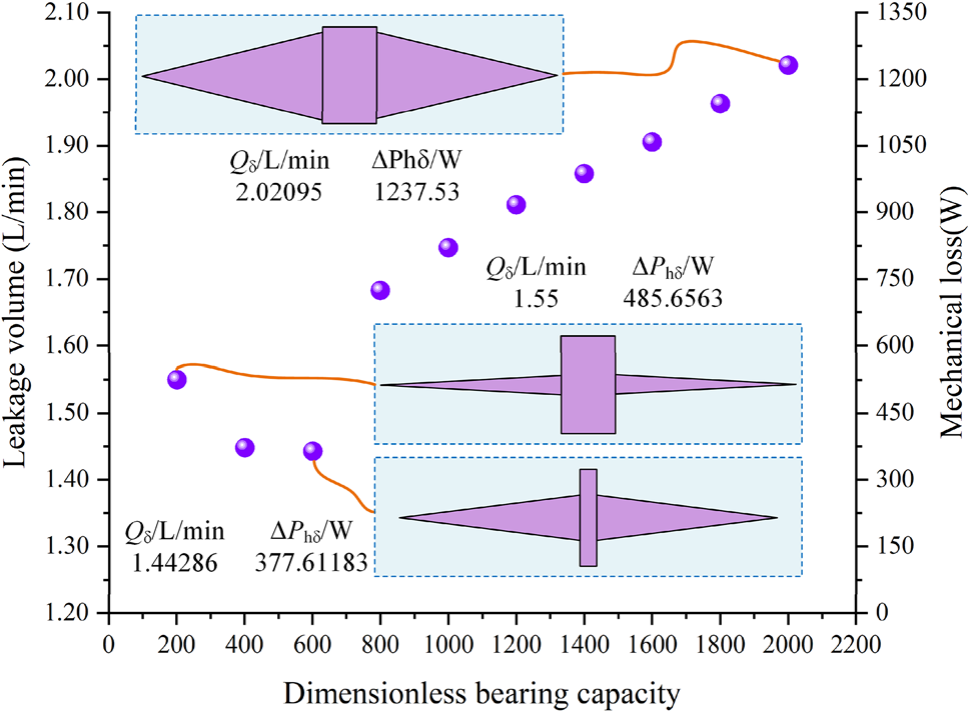
Pareto front with the optimal results.

The Pareto front of the tooth ring housing interface is shown in Fig. 11. The first result can produce the largest bearing capacity, but the mechanical loss of the first result is larger; the mechanical loss is 1237.53 W, and the leakage is 2.024 L/min. The 9th result exhibits the least mechanical loss; the mechanical loss is 377.61183 W, and the leakage is 1.44286 L/min. The second result shows that the mechanical loss is 485.6563 W and the leakage is 1.55 L/min.

## Experimental verification

### Experimental scheme

**Figure 12:**
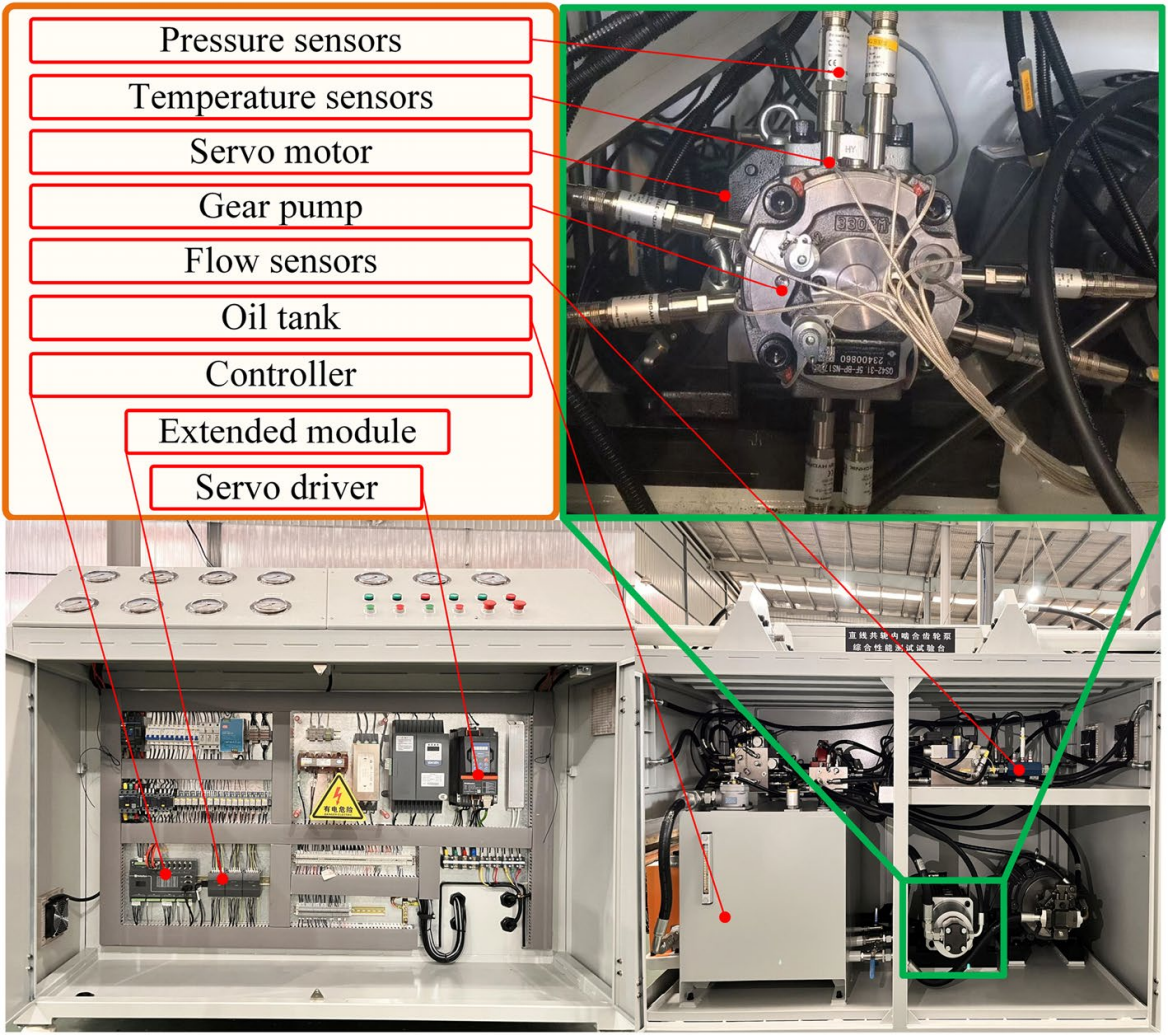
Straight-line conjugate internal meshing gear pump comprehensive performance test bench.

To verify the optimal solution of the hydrostatic bearing notch on the interface of the gear ring housing proposed in the simulation, a straight-line conjugate internal meshing gear pump test bench was built to evaluate the actual effect of the hydrostatic bearing notch. The technical parameters of key sensors on the test bench are shown in Table 4. As shown in Fig. 12, the electrical system of the test bench primarily consists of a Hollysys motion controller, a signal acquisition and expansion module, and a servo driver. The Hollysys motion controller is used to generate command signals. While the signal acquisition expansion module collects pressure, temperature, flow, and other parameters, the servo driver is used to drive the servo motor. The hydraulic system is composed of a servo motor, a hydraulic oil tank, a pressure sensor, a flow sensor, a temperature sensor, and other components. The servo motor is connected to the speed and torque sensors to drive the gear pump. Eight pressure sensors are installed at the interface of the gear ring housing to measure the pressure at that interface. Flow sensors are installed at the suction and pressure ports of the hydraulic pump, and the external leakage of hydraulic oil is measured through the measuring cup. Nine temperature sensors are installed on the interface of the gear ring housing to measure the thermal balance temperature of the gear ring interface corresponding to the surface of the housing.

**Table 4:** Test bench sensor technical parameter table.

Sensor name	Range ability	Measuring accuracy
Oil pressure port flow sensor	1–75 L/min	± 0.5% of reading
Leakage port flow sensor	0.7–20 L/min	± 0.5% of reading
Oil pressure port pressure sensor	0–250 bar	± 0.25% Fs
Oil suction pressure sensor	− 1–6 bar	± 0.25% Fs
Shell pressure sensor	0–250 bar	± 0.5% Fs
Temperature sensor	0–100 ℃	0.15 ℃
Speed torque sensor	0–100 Nm	0.3% Fs
	0–1800 r/min	1080 pulse/r

### Experimental analysis

**Figure 13:**
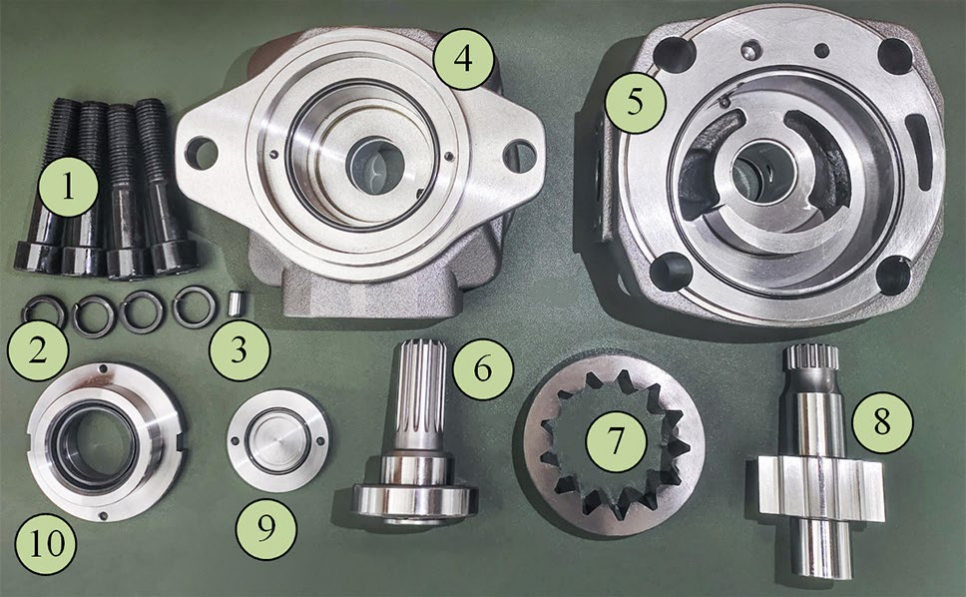
Straight-line conjugate internal meshing gear pump structure diagram.

The gear pump with an optimized hydrostatic support groove is assembled as shown in Fig. 13, where part 1 is a fixing bolt, part 2 is an elastic washer, part 3 is a positioning pin, part 4 is the front end cover of the pump, part 5 is the rear end cover of the pump, part 6 is the bearing and spline shaft, part 7 is the outer gear ring, part 8 is the internal gear, part 9 is the rear seal end cover, and part 10 is the rear shaft seal.

**Figure 14:**
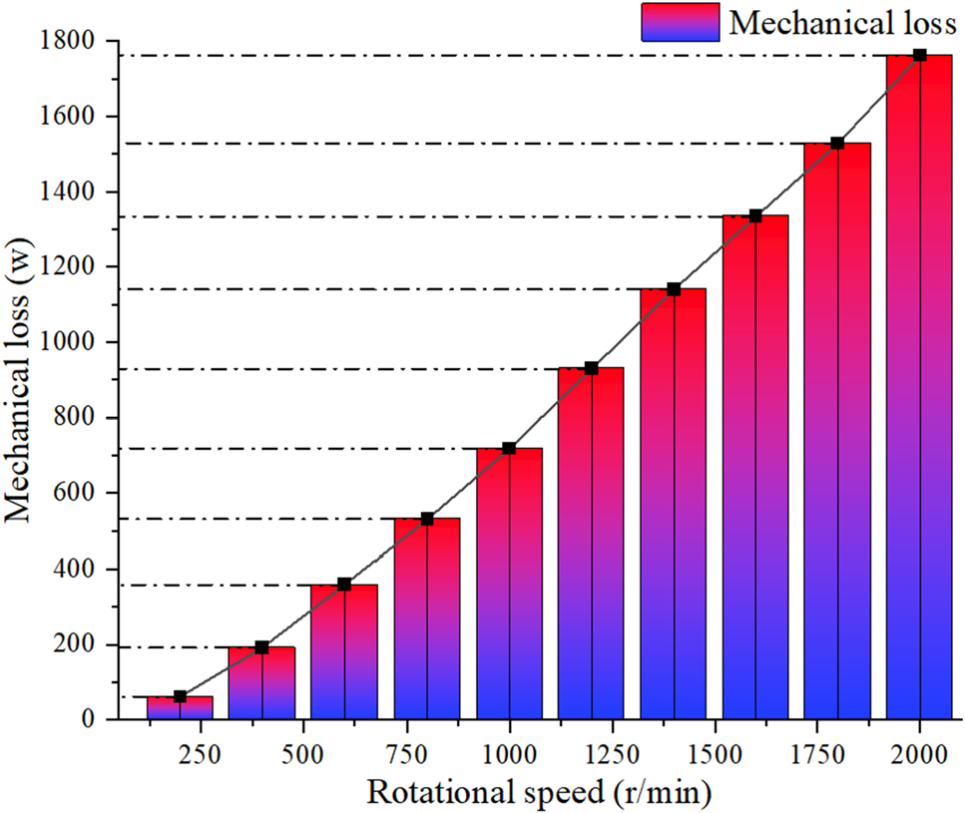
Mechanical power loss before notch optimization.

**Figure 15:**
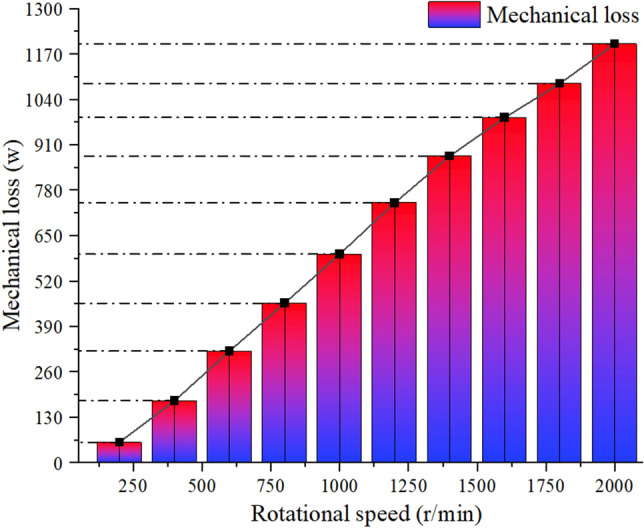
Mechanical power loss after notch optimization.

As shown in Figs. 14 and 15, after groove optimization, the overall mechanical efficiency is improved, and the higher the speed, the more obvious the mechanical loss optimization effect. Among them, the mechanical loss is reduced by 4.8% at 200 r/min, by 9.2% at 400 r/min, and by 12.5% at 600 r/min. Mechanical loss is reduced by 13.1% at 800 r/min, by 16.5% at 1000 r/min, by 19.1% at 1200 r/min, by 22.5% at 1400 r/min, and by 25.5% at 1600 r/min. The mechanical loss is reduced by 28.7% at 1800 r/min and by 31.2% at 2000 r/min.

**Figure 16:**
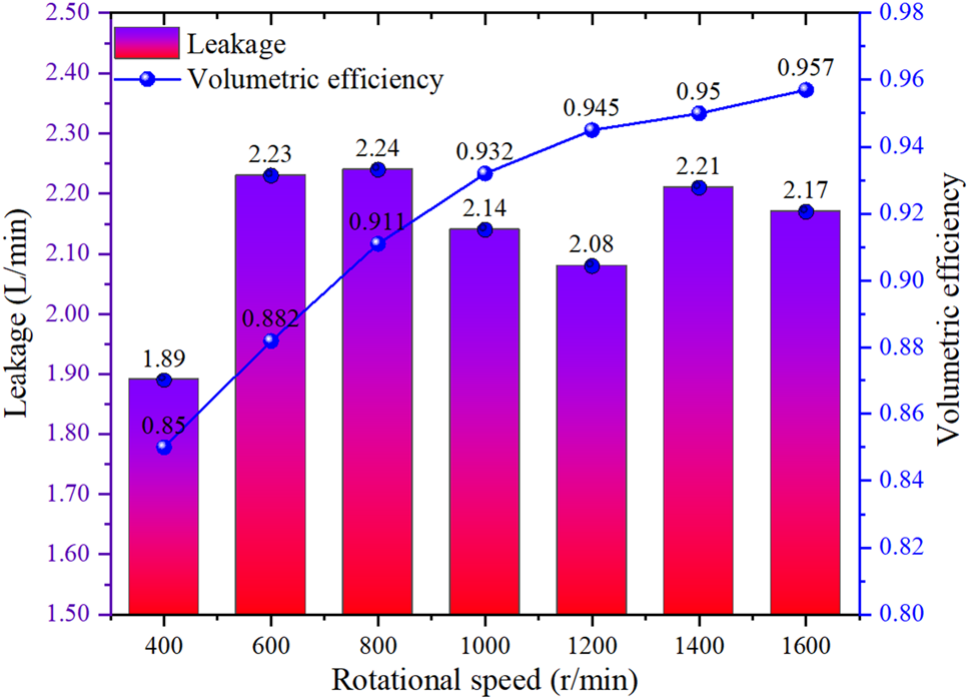
Leakage and volumetric efficiency before groove optimization.

**Figure 17:**
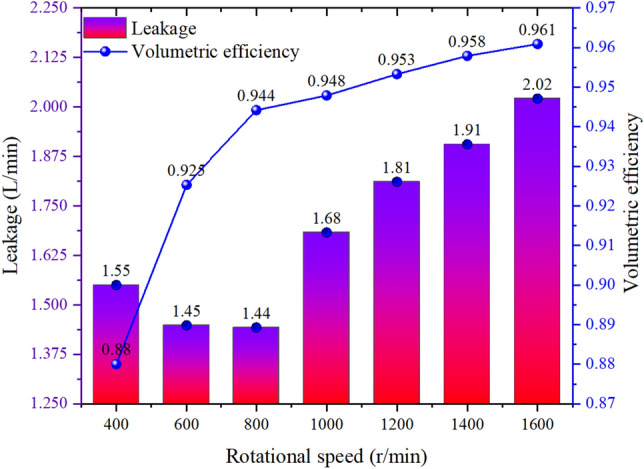
Leakage and volumetric efficiency after groove optimization.

As shown in Figs. 16 and 17, after notch optimization, the overall volumetric efficiency optimization effect increases first and then decreases with speed. Among them, the volumetric efficiency increases by 3.53% at 400 r/min, by 4.91% at 600 r/min, and by 3.64% at 800 r/min. The volumetric efficiency increases by 1.71% at 1000 r/min, by 0.88% at 1200 r/min, by 0.83% at 1400 r/min, and by 0.41% at 1600 r/min.

**Figure 18:**
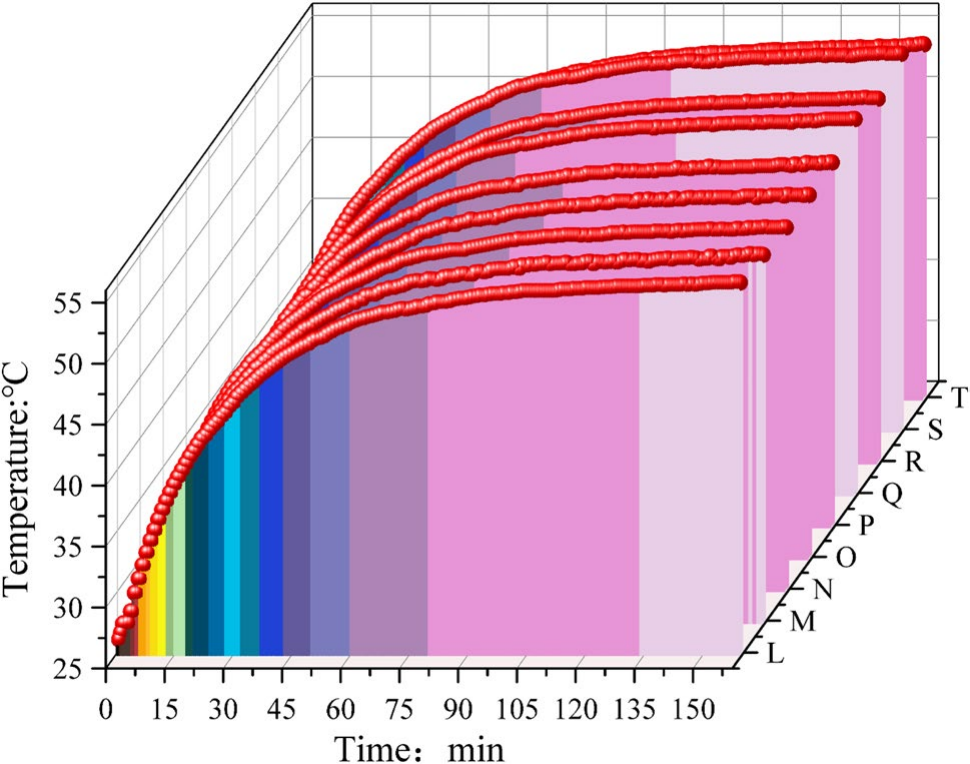
The gear ring corresponds to the heat equilibrium temperature of the outer surface of the shell.

As shown in Fig. 18, the heat balance temperature on the surface of the pump housing exhibits a trend of steep rise at first and gentle rise thereafter. Among them, the maximum heat balance temperature at point L is 54.35 °C, the maximum heat balance temperature at point M is 54.04 °C, the maximum heat balance temperature at point N is 53.63 °C, the maximum heat balance temperature at point O is 53.67 °C, and the maximum heat balance temperature at point P is 53.72 °C. The highest thermal equilibrium temperature at point Q is 54.66 °C, the highest thermal equilibrium temperature at point R is 53.77 °C, the highest thermal equilibrium temperature at point S is 54.78 °C, and the highest thermal equilibrium temperature at point T is 52.94 °C. As can be seen in Table 5, the operating temperature of the hydraulic oil does not exceed 45 °C, and the thermal equilibrium temperature of the shell does not exceed 55 °C, which is far lower than the maximum permissible operating temperature of the shell surface.

**Table 5:** Maximum allowable surface temperature table for hydraulic pump casings.

Hydraulic oil operating temperature *T*_o_ (°C)	Maximum permissible temperature of shell surface *T*_c_ (°C)
Minimum operating oil temperature ~ 30 °C	55 °C
30–45 °C	*T*_o_ + 25 °C
Maximum operating oil temperature ~ 45 °C	70 °C

## Conclusion

Through an optimizing simulation and experimental verification, the following conclusions can be obtained: a multi-dimensional, multi-objective optimization simulation model of a static pressure-bearing notch based on NSDE is proposed. The optimal Pareto solution set for the notch shape of the hydrostatic bearing is obtained. According to the multi-objective simulation optimization model, the mechanical loss of the 9th notch shape is the least; the mechanical loss is 377.61183 W, and the leakage is 1.44286 L/min. According to the test bench of the straight-line conjugate internal meshing gear pump, the 9th notch shape can reduce the mechanical loss of the straight-line conjugate internal meshing gear pump by 31.52% at 2000 r/min and increase the volumetric efficiency by 4.91% at 600 r/min. In future work, the motion path of the gear ring under various forces will be considered. The eccentricity of the gear ring housing is updated in real time to improve the model.

## Data Availability

The data that supports the findings of this study are available from the corresponding author on request.
